# Assessment of Liver Regeneration in Patients Who Have Undergone Living Donor Hepatectomy for Living Donor Liver Transplantation

**DOI:** 10.3390/vaccines11020244

**Published:** 2023-01-21

**Authors:** Basri Satilmis, Sami Akbulut, Tevfik Tolga Sahin, Yasin Dalda, Adem Tuncer, Zeynep Kucukakcali, Zeki Ogut, Sezai Yilmaz

**Affiliations:** 1 Department of Surgery and Liver Transplant Institute, Inonu University Faculty of Medicine, Malatya 244280, Turkey; 2 Department of Biochemistry, Inonu University Faculty of Pharmacy, Malatya 244280, Turkey; 3 Department of Biostatistics, and Medical Informatics, Inonu University Faculty of Medicine, Malatya 44280, Turkey

**Keywords:** liver transplantation, living donor hepatectomy, liver regeneration, liver progenitor cells

## Abstract

Background: Inflammation and the associated immune pathways are among the most important factors in liver regeneration after living donor hepatectomy. Various biomarkers, especially liver function tests, are used to show liver regeneration. The aim of this study was to evaluate the course of liver regeneration following donor hepatectomy (LDH) by routine and regeneration-related biomarkers. Method: Data from 63 living liver donors (LLDs) who underwent LDH in Inonu University Liver Transplant Institute were prospectively analyzed. Serum samples were obtained on the preoperative day and postoperative days (POD) 1, 3, 5, 10, and 21. Regenerative markers including alfa-fetoprotein (AFP), des carboxy prothrombin (DCP), ornithine decarboxylase (ODC), retinol-binding protein 4 (RBP4), and angiotensin-converting enzyme isotype II (ACEII) and liver function tests including alanine aminotransferase (ALT), aspartate aminotransferase (AST), gamma-glutamyl transferase (GGT), alkaline phosphatase (ALP) and total bilirubin levels were all analyzed. Results: The median age of the LLDs was 29.7 years and 28 LLDs were female. Eight LLDs developed postoperative complications requiring relaparotomy. The routine laboratory parameters including AST (<0.001), ALT (<0.001), ALP (<0.001), and total bilirubin (<0.001) showed a significant increase over time until postoperative day (POD) 3. For the regeneration-related parameters, except for the RBP4, all parameters including ACEII (*p* = 0.006), AFP (*p* = 0.002), DCP (*p* = 0.007), and ODC (*p* = 0.002) showed a significant increase in POD3. The regeneration parameters showed a different pattern of change. In right-lobe liver grafts, ACEII (*p* = 0.002), AFP (*p* = 0.035), and ODC (*p* = 0.001) showed a significant increase over time. DCP (*p* = 0.129) and RBP4 (*p* = 0.335) showed no significant changes in right-lobe liver grafts. Conclusions: Regenerative markers are increased in a sustained fashion following LDH. This is more prominent following right-lobe grafts which are indicative of progenitor-associated liver regeneration.

## 1. Introduction

Liver regeneration has been known for centuries and has been subject to many myths. The regenerative capacity of the liver determines the success of many surgical procedures such as anatomic and non-anatomic or living donor hepatectomy (LDH), which is an important part of living donor liver transplantation (LDLT) [1]. Furthermore, this ability of the liver protects organisms from many injurious effects [2]. There are two main liver regeneration mechanisms: typical and progenitor cell-dependent regeneration. Both mechanisms are triggered during any injury to the liver, however, the dominance of either mechanism depends on the amount of liver tissue that is affected by the injurious event. The studies on experimental models have shown that the regeneration of the liver has several stages including priming, proliferation, and termination, all providing hepatocyte proliferation and hypertrophy [3,4,5].

The process of regeneration has been studied extensively in preclinical models and factors such as the hepatocyte growth factor (HGF), interleukin-6 (IL-6), tumor necrosis factor-α (TNF- α), and signal transducer and activator of transcription 3 (STAT3), whilst Notch and Yap pathways have been found to be important in the regenerative process. During the era of COVID-19, the role of angiotensin-converting enzyme II receptors (ACEII) has been emphasized [6,7,8,9]. Studies have shown that ACEII is over-expressed early after partial hepatectomy and is sustained until the termination of the regenerative process [6,7,8,9]. Studies regarding the ACEII levels following liver resection in humans are lacking. Retinol binding protein (RBP) and especially RBP4 have been shown to play a role in glucose homeostasis in the liver in various pathological processes such as non-alcoholic fatty liver disease [10]. However, its role in the regenerating liver where hypoglycemia is prevalent has not been studied. Ornithine decarboxylase (ODC) is an important enzyme in the catabolism of ornithine to polyamines which is very important in the stabilization of the deoxyribonucleic acid (DNA) structure. ODC can be considered as an indirect marker for DNA synthesis [11]. There is a lack of literature analyzing the role of ODC in liver regeneration in liver transplantation. Alfa Feto Protein (AFP) is mainly involved in hepatoblasts and embryonic hepatic progenitor cells [12]. These cells are shown to be conditionally activated following various injurious effects in preclinical models [12]. Therefore, it can be stated that AFP is associated with hepatic progenitor cell-dependent liver regeneration such as those seen in massive hepatic resections and toxic liver injury [12]. The association of changes in AFP levels needs to be analyzed with the regenerative process in LDLT. Des-gamma-carboxy prothrombin (DCP) is a known marker for the prognostication and diagnosis of hepatocellular carcinoma [13]. However, changes in the levels of DCP in hepatic regeneration have not been analyzed before.

As can be seen clearly, there is a lack of comprehensive studies regarding the regenerative process following living donor hepatectomy (LDH) and in the recipients following the transplantation of the partial liver graft. Furthermore, the majority of our knowledge regarding liver regeneration following partial hepatectomy originates from preclinical experimental models [14].

We are a center of excellence in LDLT and we perform an average of 300 cases of LDLT annually. Therefore, in the present study, we aimed to evaluate the process of liver regeneration in living liver donors (LLDs), who underwent LDH by evaluating serum biomarkers of regeneration including AFP, DCP, ACEII, ODC, and RBP4.

## 2. Materials and Methods

### 2.1. Study Population and Design

This is a prospective cohort study. We performed a power analysis to determine the minimum number of subjects that should be included in the present study. Power analyses showed that, with the study power being 0.95, the alpha coefficient being 0.05, and the effect size being (d) = 0.5, the minimum number of subjects required for the study was calculated to be 44. The power analysis was performed using G*Power 3.1.9.7 (Düsseldorf, Germany). We prospectively analyzed and followed up with 63 LLDs who received LDH between 2020 and 2022 in Inonu University Liver Transplant Institute and included in our study. Our LLDs evaluation algorithm, LDH technique, and postoperative follow-up protocol have been previously defined [15]. Verbal and written informed consent were obtained from all LLDs.

### 2.2. Study Parameters

The demographic characteristics including age, sex, body mass index (BMI), habits (smoking and alcohol consumption), blood type (ABO and Rh), operative parameters including the type of liver graft resected, graft weight, remnant liver volume, postoperative complications requiring relaparotomy (such as bleeding and biliary peritonitis) and clinical variables including the routine laboratory parameters on preoperative and postoperative days (PODs) 1, 3, 5, 7, 10 and 21 were collected. Specific laboratory values including alfa-fetoprotein (AFP), des carboxy prothrombin (DCP), ornithine decarboxylase (ODC), retinol binding protein 4 (RBP4), and angiotensin converting enzyme isotype II (ACEII) levels were measured in the same time intervals.

All routine laboratory evaluations including liver function tests, complete blood count, and coagulation studies were obtained from the hospital database. The demographic and operative parameters were obtained from the electronic patient registry. Five milliliters of extra blood was drawn from the subjects and transferred to Inonu University Liver Transplant Institute Hepatology Research Laboratories and all the samples were centrifuged at 2000 rpm at 4 °C for 10 min and serum obtained from the blood samples were divided into four aliquots and stored at −80 °C until the experiments were performed. The comparison of these variables was performed according to different study subgroups; (i) the routine and specific laboratory variables were compared according to sex (male versus females); (ii) according to LDH type (right-lobe LDH versus left-lobe LDH (including left-lobe lateral segment)); and (iii) according to the presence or absence of complications requiring relaparotomy.

### 2.3. Measurement of the Specific Laboratory parameters

All routine and specific biochemical analysis were performed at Inonu University Liver Transplant Institute Hepatology Research Laboratories. All specific measurements including AFP, DCP, ACEII, ODC, and RBP4 were performed using enzyme-linked immunosorbent assay (ELISA). The measurements were performed by using Human AFP ELISA Kit (BT-LAB, Cat No: E1630Hu), Human DCP ELISA Kit (BT-LAB, Cat No: E4012Hu), Human ACEII ELISA Kit (BT-LAB, Cat No: E3169Hu), Human ODC ELISA Kit (BT-LAB, Cat No: E0845Hu), and Human RBP4 ELISA Kit (BT-LAB, Cat No: E1206Hu) according to manufacturer’s instructions. For all ELISA measurements, after pipetting 40 µL of serum samples to the wells of the precoated ELISA plate, 10 µL of the biotinylated antibody of AFP, DCP, ACEII, ODC, and RBP4 were added to sample wells, respectively. Then, 50 µL of Streptavidin-HRP was added and incubated for 1 h at 37 °C. At the end of the incubation period, the wells were washed thoroughly, 50 µL of both Solution A and B were added and incubated for 10 min at 37 °C in the dark. Then, 50 µL of Stop Solution was pipetted and the color of the solution turned yellow. Absorbance was determined by using Biotek Synergy H1m™ microplate reader (BioTek Instruments Inc., Winooski, VT, USA) at 450 nm within 10 min.

### 2.4. Ethics Committee Approval and Financial Support

The ethics committee approval was obtained from the Malatya Clinical Research Ethics Committee (Approval no: 2020/170). All stages of the study were carried out according to the guidelines of the Declaration of Helsinki. The financial support was received from the Inonu University Scientific Research Projects Coordination Unit (Project ID: TSA-2021-2382).

### 2.5. Statistical Analysis

The categorical variables were expressed as the number of individuals and percentage of the study population. Continuous variables are expressed as mean ± standard deviation or median (95% lower CL for median; 95% upper CL for median). The normality of continuous variables was tested using the Kolmogorov–Smirnov and Shapiro–Wilk tests. The Friedman, Mann–Whitney U, and Kruskal–Wallis tests were used to compare the dependent variables among the study groups. Any *p*-value less than 0.05 was considered statistically significant. All analyses were performed using the Statistical Software Package for Social Sciences version 26.0 (IBM, SPSSv26.0, Armonk, NY, USA).

## 3. Results

### 3.1. General Assessment of Demographic and Clinical Variables

In total, 63 LLDs who received LDH were included in the present study. The median age of the LLDs were 29 (95% CI = 28–34) years. There were 28 female (44.4%) and 35 male (55.6%) subjects. The median BMI of the LLDs was 24.7 kg/m^2^ (95% CI = 23.3–25.9). The blood groups of the LLDs were group 0 in 33 (52.4%), group A in 20 (31.8%), and group B in 10 (15.9%) subjects. Twenty (31.8%) LLDs were smokers and five (7.9%) consumed alcohol on regular basis. Right-lobe LDH was performed in 43 subjects (68.2%), and the left-lobe LDH was performed in 10 LLDs (15.9%), and left lateral segmentectomy was performed in 10 (15.9%) subjects. The median future remnant liver volume of the LLDs was 32 (95% CI = 32–35). The median liver graft volume was 700 cc 95% CI = 670–770). Eight LLDs (12.7%) suffered from early perioperative complications requiring emergency laparotomy. We observed no postoperative mortality in any of the LLDs. The demographic and some clinical variables are summarized in Table 1.

### 3.2. Analysis of the Biochemical Parameters in Whole Study Group

#### 3.2.1. Change of the Routine Biochemical Parameters

**Figure 1:**
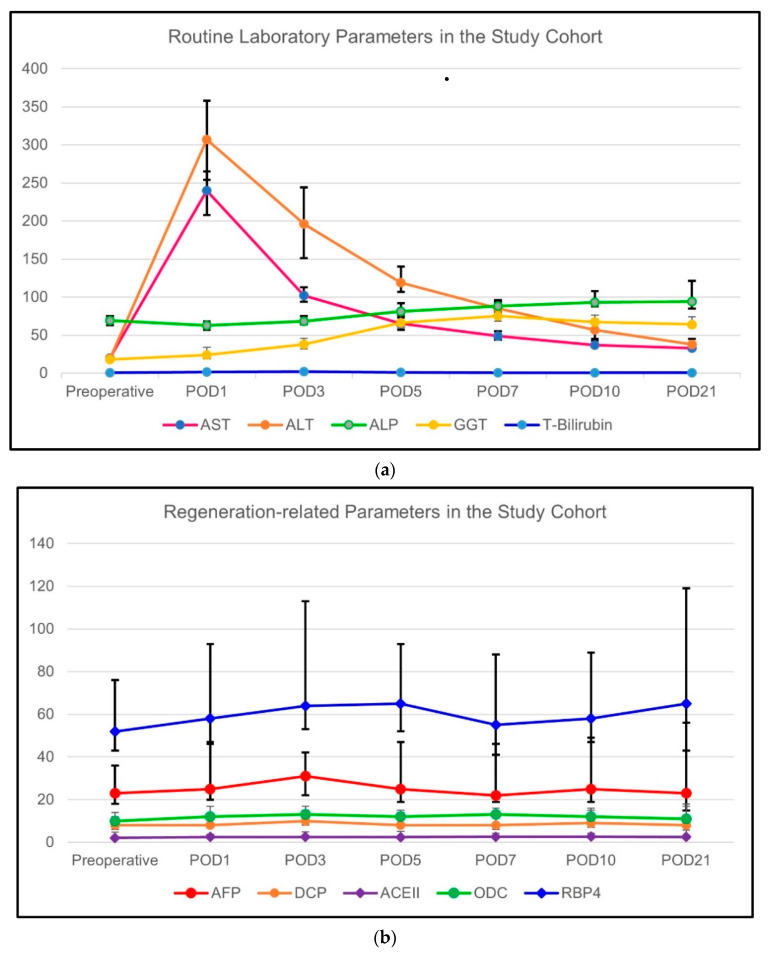
(**a**) The course of routine biochemical blood parameters of the LLDs in the study group; and (**b**) the course of regeneration-related biochemical parameters of the LLDs in the study group.

The changes in routine biochemical markers over the designated time period are summarized in Table 2. These biochemical results are also graphically shown in Figure 1a,b. The routine laboratory parameters including AST (<0.001), ALT (<0.001), ALP (<0.001), and total bilirubin (<0.001) of the whole study cohort showed a significant increase over time starting from the preoperative period until the postoperative day (POD) 3. Thereafter, the routine laboratory values returned to the normal range until the POD21. The only exception was the GGT (<0.001) which remained elevated starting from the POD1 until the end of the study period.

#### 3.2.2. Change of Regeneration-Related Biochemical Parameters

The course of regeneration-related biochemical markers over the designated time period is summarized in Table 2. For regeneration-related parameters, except for the RBP4, all parameters including ACEII (*p* = 0.006), AFP (*p* = 0.002), DCP (*p* = 0.007), and ODC (*p* = 0.002) showed a modest increase in POD3 which was statistically significant. In RBP4 (*p* = 0.084), there was a tendency towards an increase in the serum levels in POD 3 but it did not reach statistical significance.

### 3.3. Analysis of the Biochemical Parameters in Selected Study Subgroups

#### 3.3.1. Comparison of LLDs with Right-Lobe versus Left-Lobe LDH

##### Change of the Routine Biochemical Parameters

**Figure 2:**
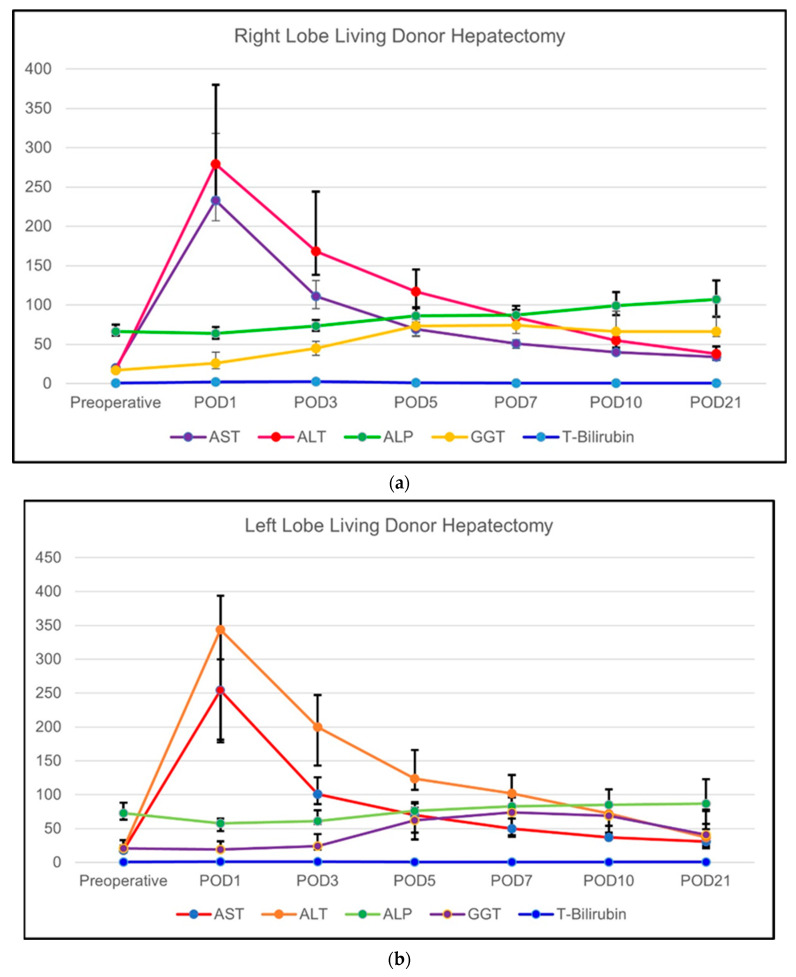
(**a**) The course of routine biochemical blood parameters of LLDs who underwent right-lobe LDH; and (**b**) the course of routine biochemical blood parameters of LLDs who underwent left (including left lateral segment)-lobe LDH.

The changes in routine biochemical markers over the designated time period among subgroups (right-lobe LDH versus left-lobe LDH) are summarized in Table 3. These biochemical results are also graphically shown in Figure 2a,b. The type of LDH did not have a significant effect on the routine laboratory values in AST, ALT, GGT, and ALP at any time point. The only exception was AST in the POD5 (*p* = 0.034) and POD10 (*p* = 0.039) which were significantly higher in the right-lobe when compared to left-lobe grafts. However, the total bilirubin was significantly higher in the LLDs who underwent right-lobe LDH in POD1 (<0.001), POD3 (<0.001), POD5 (<0.001), POD7 (<0.001), POD10 (<0.001), and POD21 (*p* = 0.004). Similarly, the group analysis of the routine parameters in the individual subgroups showed a similar pattern of changes as in the general population.

##### Change of the Regeneration-Related Biochemical Parameters

**Figure 3:**
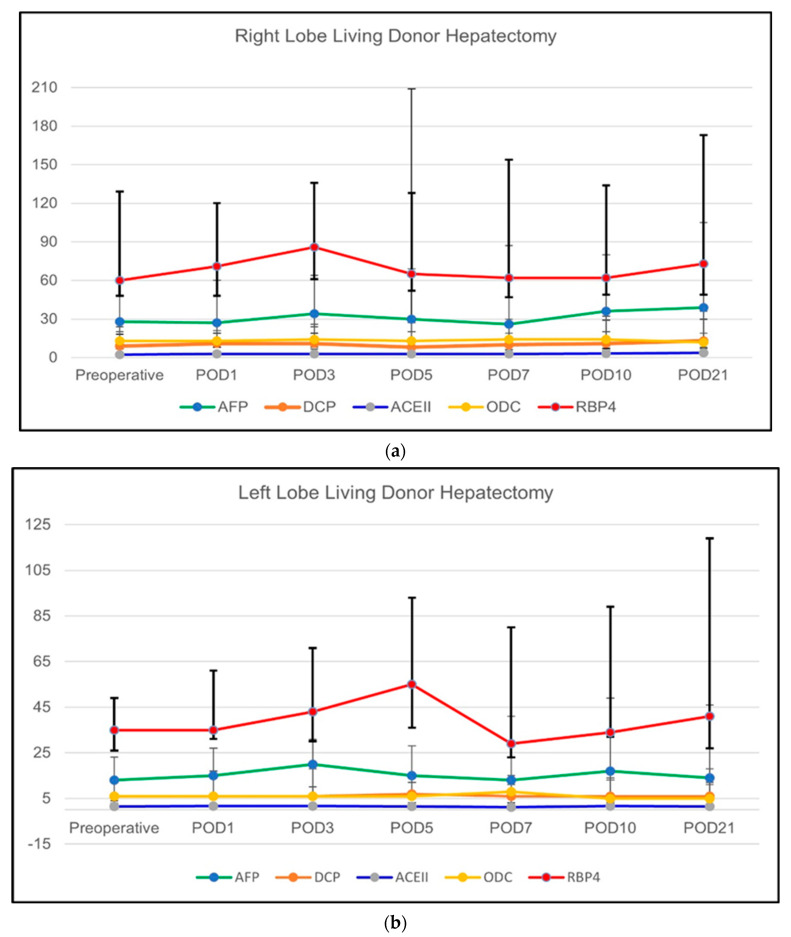
(**a**) The course of regeneration-related biochemical parameters of LLDs who underwent right-lobe LDH. (**b**) The course of regeneration-related biochemical parameters of LLDs who underwent left (including left lateral segment)-lobe LDH.

The course of regeneration-related biochemical markers over the designated time period among subgroups (right-lobe LDH versus left-lobe LDH) are summarized in Table 4. These biochemical results are also graphically shown in Figure 3a,b. The regeneration-related parameters showed a different pattern of change. In the right-lobe LDH subgroup, ACEII (*p* = 0.002), AFP (*p* = 0.035), and ODC (*p* = 0.001) showed a significant increase starting from the POD1 until the POD21. DCP (*p* = 0.129) and RBP (*p* = 0.335) showed no significant changes in the right-lobe LDH subgroup. On the other hand, only DCP showed a significant increase in the left-lobe LDH subgroup throughout the designated time periods (*p* = 0.035). The comparison between the right-lobe LDH and left-lobe LDH groups showed that ACEII was significantly higher in the right-lobe LDH group during all time periods except POD10 (*p* = 0.058). Similarly, AFP was significantly higher in all time periods in the right-lobe LDH group except for the POD10 (*p* = 0.079). The comparison of DCP showed that the right-lobe LDH group had significantly higher levels during the preoperative period (*p* = 0.010) and POD1 (*p* = 0.008), POD3 (*p* = 0.021), and POD7 (*p* = 0.008). RBP was significantly higher in right-lobe LDH group during the preoperative period (*p* = 0.044), POD1 (*p* = 0.022), POD3(*p* = 0.015), POD7 (*p* = 0.009), and POD21 (*p* = 0.039). The ODC was significantly higher in the right-lobe LDH group in preoperative period (*p* = 0.05) and POD5 (*p* = 0.021), POD10 (*p* = 0.02), and POD21 (*p* = 0.015).

#### 3.3.2. Comparison of LLDs with and without Postoperative Complications

##### Change of the Routine Biochemical Parameters

**Figure 4:**
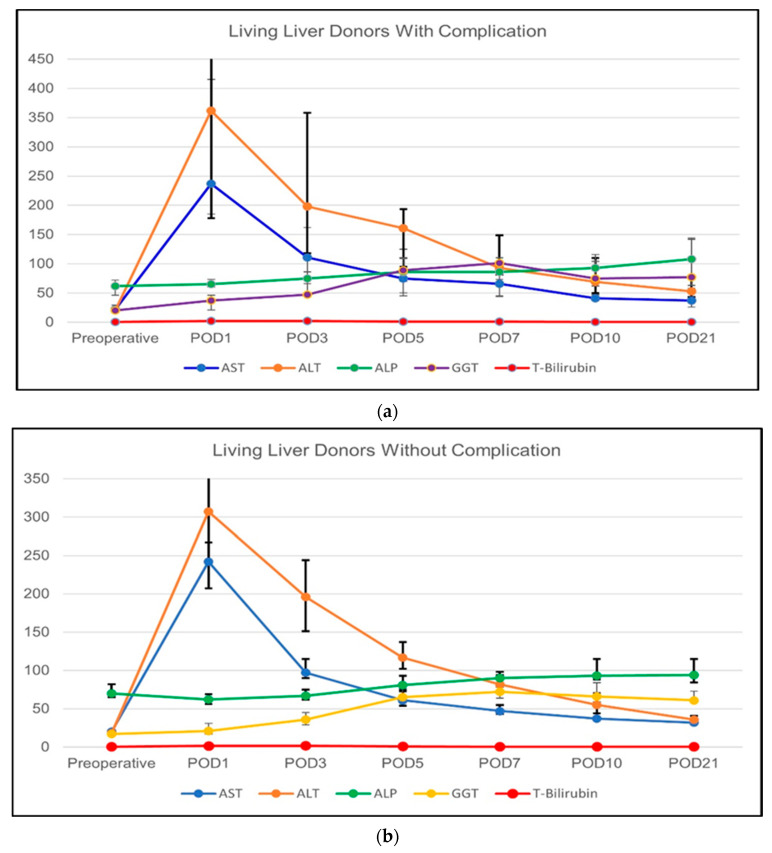
(**a**) The course of routine biochemical blood parameters of LLDs with postoperative complications (requiring relaparotomy); and (**b**) the course of routine biochemical blood parameters of LLDs without postoperative complications.

The routine biochemical markers of LLDs with and without complication were compared and the obtained results are given in Table 5. In LLDs with postoperative complications, the change of AST (*p* < 0.001), ALT (*p* < 0.001), GGT (*p* < 0.001), ALP (*p* < 0.001), and total bilirubin (*p* < 0.001) significantly increased over time towards POD5 and then returned to normal ranges. Similarly, LLDs without postoperative complications showed the same trend of change in AST (*p* < 0.001), ALT (*p* < 0.001), GGT (*p* < 0.001), ALP (*p* < 0.001), and total bilirubin (*p* < 0.001). When the two subgroups were compared in terms of these routine biochemical markers, there seemed to be no difference in most of the parameters at any time point. However, the total bilirubin was slightly higher in the LLDs with complications in POD7 (*p* = 0.017) and POD21 (*p* = 0.009). The results of these analyses are summarized in Figure 4a,b.

##### Change of the Regeneration-Related Biochemical Parameters

**Figure 5:**
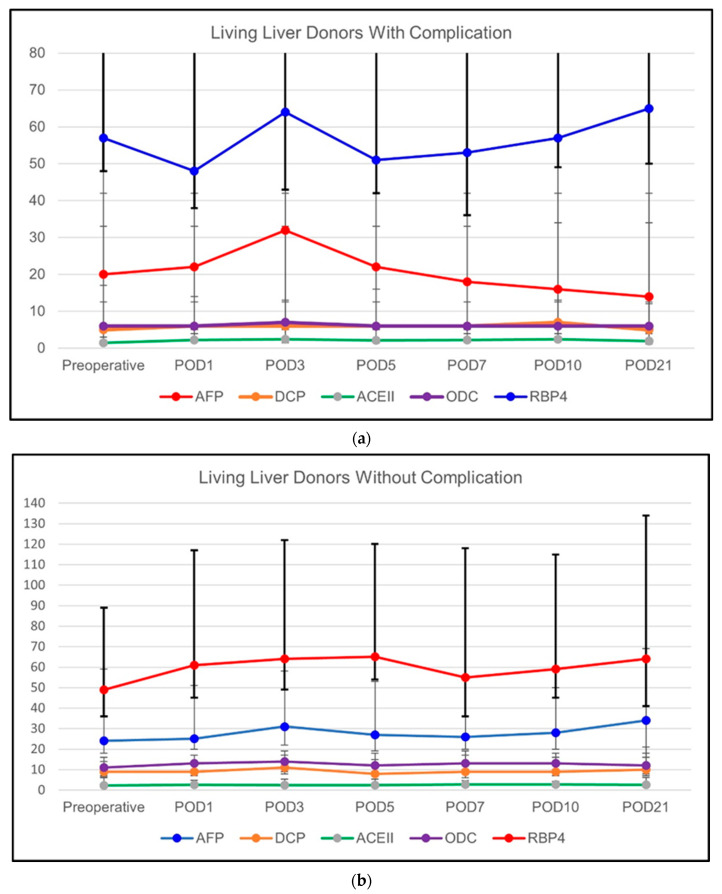
(**a**) The course of regeneration-related biochemical parameters of LLDs with postoperative complications (requiring relaparotomy); and (**b**) the course of regeneration-related biochemical parameters of LLDs without postoperative complications.

The regeneration-related biochemical markers were also compared among the LLDs with and without postoperative complications and the results are given in Table 6. In LLDs with postoperative complications, we did not observe any significant difference over time in any of these parameters. In LLDs without postoperative complications, ACEII (*p* = 0.027), AFP (*p* = 0.003), DCP (*p* = 0.026), and ODC (*p* = 0.027) showed a slight increase over time which was statistically significant. RBP showed a trend towards an increase but did not reach significance in LLDs without complications. The comparison of the two subgroups did not show any significant difference at any time point. The results of the analyses are summarized in Figure 5a,b.

#### 3.3.3. Comparison of LLDs by Sex

In our study, there were 28 female LLDs and 8 of these (28.5%) underwent left- or left-lateral-lobe donor hepatectomy. There were 35 male LLDs and 12 (35.3%) that underwent left- or left-lateral-segment LDH. There were no significant differences in terms of donor hepatectomy types between the two groups (*p* = 0.47).

##### Change of the Routine Biochemical Parameters

Similar to the general population, in the female patients, serum AST (*p* < 0.001), ALT (*p* < 0.001), GGT (*p* < 0.001), ALP (*p* < 0.001), and TBil (*p* < 0.001) levels showed a steady and significant increase starting from the POD1 until POD 5 and then steadily decreased to normal levels throughout the rest of the follow-up period. Similar results were obtained in the male LLDs in the course of serum levels of AST (*p* < 0.001), ALT (*p* < 0.001), GGT (*p* < 0.001), ALP (*p* < 0.001), and TBil (*p* < 0.001). We analyzed the course of serum levels of liver function tests within and among the sex subgroups in the designated time intervals. Only AST and ALT showed changes between the male and female LLDs. The serum AST levels of the male LLDs were significantly higher than the female LLDs on POD7 (*p* = 0.019) and POD21 (*p* = 0.035). Serum ALT values were significantly higher in the male donor on POD1 (*p* = 0.040), POD7 (*p* = 0.012), POD10 (*p* = 0.005), and POD21(*p* < 0.001). There were no significant differences in the remaining liver function tests between the male and female LLDs. The comparisons of the liver functions tests within the sex groups and between the male and female patients are summarized in Table 7.

##### Change of the Regeneration-Related Biochemical Parameters

The changes in the serum levels of the regeneration-related biochemical parameters were analyzed according to the sex of the LLDs. The results of the analyses of the regeneration-related biochemical parameters according to the sex of the LLDs are summarized in Table 8. In female LLDs, changes in the serum levels of DCP (*p* = 0.004) and ODC (*p* = 0.015) showed a significant and sustained increase in designated time intervals. On the other hand, the serum levels of ACEII, AFP and RBP did not show a significant change between the different time intervals, although there is a tendency for all three parameters to show a sustained increase in the postoperative periods.

On the other hand, male patients showed a sustained and significant increase in serum levels of ACEII (*p* = 0.027) and AFP(*p* = 0.025) throughout the follow-up period. RBP, DCP, and ODC showed no significant change in the designated time intervals in male patients. The comparison of the regeneration-related biomarkers among the two sexes did not show any difference in any time interval.

#### 3.3.4. Comparison of LLDs in Terms of Remnant Liver Volume (RLV) (%)

LLDs were divided into three subgroups based on the percentage of RLV: RLV≤ 30%, RLV = 31–35% and RLV ≥ 36%. The groups were compared in terms of changes in regeneration-related biochemical parameters and the results are summarized in Table 9.

## 4. Discussion

Liver regeneration is a crucial component that determines the success of major hepatic resections such as formal hepatectomy, informal hepatectomy, and LDH. The regenerative capacity of the liver enables us to perform major liver resections and if this regenerative process is disturbed in either way, post-hepatectomy liver failure or a small-for-size syndrome is observed [16]. After major hepatectomy series, the incidence of post-hepatectomy liver failure is reported as high as 32% [17]. In the present study, we have found that markers of regeneration change significantly after LDH in LLDs. This phenomenon is more prominent in right-lobe liver grafts. The change in the serum levels of the markers is not prominently observed in LLDs with complication who require re-laparotomy. To our knowledge, this is the only study analyzing the markers of regeneration in LLDs.

Conventional markers for liver and hepatocyte damage are ALT, AST, and ALP [18]. However, these are not sufficient to evaluate the regenerative process of the liver [19]. There is clearly a need for additional markers to evaluate the regenerative capacity of the liver. Furthermore, there is a need for the evaluation of the normal postoperative course of these markers so that the deviation from the normal course can be evaluated under complicated conditions. In the present study, the markers of hepatocellular markers such as AST, ALT, ALP, and GGT have been elevated towards the POD3 and POD5. Thereafter, a decline in the levels of these enzymes has been observed. This pattern of change was observed in both right, left, and left lateral liver grafts. However, in terms of remnant liver volume, right-lobe liver grafts are associated with lower remnant liver volumes when compared to [20]. This has been reflected in the postoperative course of the enzymes and AST, ALT, ALP, and GGT have been significantly higher in the right-lobe grafts in various postoperative time intervals when compared to left- and left-lateral-liver grafts. Similarly, ALT and AST levels of the male LLDs were higher than the female in postoperative periods. This may be related with the liver mass and the amount of liver tissue resected from the male patients. However, we could not find any data in the literature to explain the differences in liver function tests in LLDs according to sex.

We previously found that the total bilirubin was an important factor in determining the prognosis of the patient’s acute liver failure who has undergone liver transplantation [21]. Studies have shown that the bilirubin levels are indicative of the function of the liver and it is correlated with the severity of liver dysfunction [22,23]. In the present study, we found that the total bilirubin levels of the right-lobe liver grafts have been significantly higher than the other liver grafts which may be related with the larger mass of the liver removed and the associated decrease in the function initially observed in LLDs. Furthermore, in complicated patients, the postoperative bilirubin levels tended to be higher during the postoperative period, although this was not statistically significant. However, this finding shows the impact of any postoperative period on the function of the liver.

Historically, Rao et al. [24] showed that, in chloroform poisoning, serum markers such as GGT, DCP, and AFP were elevated and their combined analysis with the markers of hepatocellular damage would give accurate prognostic information for the affected patients. Later on, the same team evaluated the prognostic significance of these markers in mushroom poisoning [18]. Both studies have shown that, in patients who survived, regenerative markers increased gradually while the markers of liver damage decreased. Living donor hepatectomy and especially harvesting right-lobe grafts has a risk of post-hepatectomy liver failure [25]. Furthermore, there is a certain level of liver dysfunction in the early postoperative period following living donor hepatectomy [26]. For this reason, we wanted to evaluate the course of regenerative markers and the markers of hepatocellular damage in LLDs. We found that the markers of hepatocellular damage have gradually increased towards the POD3 and POD5 and gradually decreased thereafter. On the other hand, we observed a sustained elevation of AFP, DCP, ACEII, ODC, and GGT. Furthermore, this trend in the levels of regeneration markers did not occur in left-lobe liver grafts (except for DCP elevation); however, the right-lobe liver grafts showed a pattern of sustained AFP, DCP, ACEII, and ODC elevation. This proves that, after the removal of right-lobe liver grafts, progenitor oval cells actively take part in the regeneration of the remnant liver. The data regarding the serum ACEII levels following the living donor hepatectomy is lacking. Our study is the first study to emphasize the regeneration-related role of ACEII and it is mainly enhanced in right-lobe liver grafts. Furthermore, our study showed a sex difference between the serum levels of ACEII, which was previously studied in patients with hypertension and cardiac failure [27,28]; however, not in a liver transplant setting. The results of the present study show that there is a difference between the regenerative capacity of the female and male LLDs. It has been emphasized in a limited number of studies that the livers of female patients have a higher regenerative potential [29,30,31]. Unfortunately, our data are limited in terms of their ability to explain this observation, however, we can clearly state that it is not related to the type of graft because the frequency of the right- and left-lobe liver grafts were similar among the groups. The evaluation of the regeneration-related biomarkers and their changes according to the sex of the LLDs requires further studies with higher patient numbers.

However, in left and left lateral liver grafts, the changes in the levels of the enzymes AST, ALT, ALP, and GGT were like the general population. Therefore, evaluating the regenerative process according to elevated liver enzymes may be misleading. The DCP levels in left-lobe liver grafts changed mildly but it was statistically significant. Although our literature search did not yield any relationship between DCP and progenitor cells, our results also suggest that DCP may be indicative of the hepatocyte activity after partial hepatectomy rather than a progenitor cell marker. AFP, ACEII, and ODC seem to be better markers for progenitor-assisted liver regeneration.

We only had eight patients with early postoperative complications requiring relaparotomy. The regenerative markers tend to be elevated in these patients and the fluctuations in the level of the liver enzymes were similar to the general population. We believe that we did not observe any significant changes in the serum levels of the regenerative markers because there was a small group of complicated LLDs.

Our study is the first study to address liver regeneration in LLDs. Furthermore, it gives valuable information regarding the changes in the levels of these biomarkers and the transaminases. However, the current study has some limitations. The first one is the low number of patients. Although we reached the minimum number of patients calculated in a power analysis, the regenerative markers showed wide variation. Therefore, we believe that studies with a larger number of patients will reduce the margin of error. Furthermore, we tried to perform CT volumetry as a part of the follow-up of these LLDs; however, the compliance of the LLDs was low and there were a lot of missing data. Performing volumetric analysis and correlating the results with the levels of the regenerative markers would yield valuable information.

In conclusion, there is dual hepatic regeneration and the dominant mechanism depends on the volume of the resected liver. In right-lobe liver grafts, the remnant is small and this triggers progenitor cell-mediated regeneration. However, in left-lobe and left-lateral-lobe grafts, progenitor-related regenerative markers are not elevated. AFP, ACEII, and ODC are good markers for the surveillance of regeneration following living donor hepatectomy.

## Figures and Tables

**Table 1:** Demographic and some clinical data of the LLDs included in the study group.

Variables	Results
Age (Median (95% CI))	29 (28–34)
BMI (Median (95% CI))	24.7 (23.3–25.9)
Graft volume (Median (95% CI))	
Volume (Overall)	700 (670–770)
Volume (Right-lobe graft)	770 (740–850)
Volume (Left-lobe)	530 (400–690)
Volume (Left-lobe lateral segment)	300 (280–330)
RLV(%)(Median (95% CI))	32 (32–35)
Sex (*n*;%)	
Male	28 (44.4)
Female	35 (55.6)
Blood groups (*n*;%)	
0	33 (52.4)
A	20 (31.8)
B	10 (15.9)
Smoking (*n*;%)	
Yes	20 (31.8)
No	43 (68.2)
Alcohol use (*n*;%)	
Yes	5 (7.9)
No	58 (92.1)
Donor hepatectomy (*n*;%)	
Right lobe	43 (68.2)
Left lobe	10 (15.9)
Left lateral segment	10 (15.9)
Complications (*n*;%)	
Yes	8 (12.7)
No	55 (87.3)

**Table 2:** Routine and regeneration-related biochemical parameters of the LLDs included in the study group.

Variables (Median (95% CI))	Results	Variables (Median (95% CI))	Results
ACEII Preop	2.1 (1.7–4.7)	AST Preop	20 (19–22)
ACEII POD1	2.5 (1.9–3.7)	AST POD1	240 (208–265)
ACEII POD3	2.5 (2.3–4.9)	AST POD3	102 (94–113)
ACEII POD5	2.4 (2.1–5.1)	AST POD5	65 (57–73)
ACEII POD7	2.6 (1.9–4.2)	AST POD7	49 (44–55)
ACEII POD10	2.7 (2.1–4.2)	AST POD10	37 (37–43)
ACEII POD21	2.5 (1.6–5.7)	AST POD21	33 (31–36)
p **	0.006	p **	<0.001
AFP Preop	23 (18–36)	ALT Preop	19 (17–21)
AFP POD1	25 (20–47)	ALT POD1	307 (254–358)
AFP POD3	31 (22–42)	ALT POD3	196 (151–244)
AFP POD5	25 (19–47)	ALT POD5	119 (107–140)
AFP POD7	22 (19–46)	ALT POD7	85 (81–94)
AFP POD10	25 (19–49)	ALT POD10	57 (45–70)
AFP POD21	23 (15–56)	ALT POD21	38 (32–45)
p **	0.002	p **	<0.001
DCP Preop	8 (6–12)	GGT Preop	18 (17–22)
DCP POD1	8 (7–13)	GGT POD1	24 (19–34)
DCP POD3	10 (8–15)	GGT POD3	38 (32–46)
DCP POD5	8 (7–14)	GGT POD5	66 (60–76)
DCP POD7	8 (6–12)	GGT POD7	75 (68–83)
DCP POD10	9 (7–15)	GGT POD10	67 (60–76)
DCP POD21	8 (7–18)	GGT POD21	64 (60–74)
p **	0.007	p **	<0.001
RBP Preop	52 (43–76)	ALP Preop	69 (63–75)
RBP POD1	58 (46–93)	ALP POD1	63 (57–68)
RBP POD3	64 (53–113)	ALP POD3	68 (64–75)
RBP POD5	65 (52–93)	ALP POD5	81 (76–92)
RBP POD7	55 (41–88)	ALP POD7	88 (83–96)
RBP POD10	58 (47–89)	ALP POD10	93 (88–108)
RBP POD21	65 (43–119)	ALP POD21	94 (85–121)
p **	0.084	p **	<0.001
ODC Preop	10 (6–14)	TBil Preop	0.5 (0.5–0.6)
ODC POD1	12 (7–17)	TBil POD1	1.8 (1.5–2.2)
ODC POD3	13 (8–17)	TBil POD3	1.9 (1.6–2.6)
ODC POD5	12 (9–15)	TBil POD5	0.9 (0.8–1.2)
ODC POD7	13 (8–16)	TBil POD7	0.6 (0.6–0.8)
ODC POD10	12 (7–16)	TBil POD10	0.5 (0.4–0.7)
ODC POD21	11 (7–17)	TBil POD21	0.5 (0.5–0.6)
p **	0.002	p **	<0.001

** Friedman test.

**Table 3:** Comparison of study subgroups created according to type of hepatectomy (right-lobe LDH vs. left-lobe LDH) type in terms of routine biochemical parameters.

Variables (Median (95% CI))	Type of LDH		p *
	Right Lobe	Left Lobe ***	
AST Preop	20 (19–22)	20 (16–22)	0.976
AST POD1	233 (207–318)	246 (192–270)	0.935
AST POD3	111 (95–131)	95 (77–115)	0.163
AST POD5	69 (61–78)	53 (43–81)	0.034
AST POD7	51 (45–56)	44 (38–58)	0.674
AST POD10	40 (37–47)	35 (28–40)	0.039
AST POD21	34 (32–37)	28 (23–36)	0.083
p **	<0.001	<0.001	
ALT Preop	17 (15–21)	20 (19–28)	0.236
ALT POD1	279 (236–380)	344 (244–394)	0.570
ALT POD3	168 (138–244)	213 (155–272)	0.136
ALT POD5	117 (97–145)	125 (107–166)	0.330
ALT POD7	84 (75–94)	88 (69–114)	0.333
ALT POD10	55 (45–67)	67 (39–86)	0.794
ALT POD21	38 (30–47)	38 (21–53)	0.914
p **	<0.001	<0.001	
GGT Preop	17 (15–20)	19 (17–27)	0.090
GGT POD1	26 (19–40)	20 (16–31)	0.315
GGT POD3	45 (36–54)	28 (23–41)	0.045
GGT POD5	73 (60–81)	64 (41–76)	0.396
GGT POD7	74 (64–86)	77 (55–85)	0.785
GGT POD10	66 (60–92)	71 (57–84)	0.725
GGT POD21	66 (60–80)	60 (27–73)	0.050
p **	<0.001	<0.001	
ALP Preop	66 (61–75)	74 (59–86)	0.337
ALP POD1	64 (57–72)	61 (52–68)	0.451
ALP POD3	73 (67–81)	62 (59–70)	0.043
ALP POD5	86 (75–96)	77 (75–92)	0.425
ALP POD7	87 (75–99)	92 (73–100)	0.959
ALP POD10	99 (87–116)	92 (82–119)	0.497
ALP POD21	107 (85–131)	87 (78–107)	0.199
p **	<0.001	<0.001	
TBil Preop	0.5 (0.5–0.7)	0.5 (0.5–0.6)	0.629
TBil POD1	2.2 (1.8–2.5)	1.1 (1.0–1.3)	<0.001
TBil POD3	2.5 (2.0–3.1)	1.0 (0.9–1.2)	<0.001
TBil POD5	1.1 (0.9–1.4)	0.6 (0.5–0.7)	<0.001
TBil POD7	0.8 (0.7–1.0)	0.4 (0.4–0.5)	<0.001
TBil POD10	0.7 (0.5–0.8)	0.3 (0.3–0.5)	<0.001
TBil POD21	0.5 (0.5–0.6)	0.4 (0.4–0.6)	0.004
p **	<0.001	<0.001	

* Mann–Whitney U test; ** Friedman test; *** Left or left-lob lateral segment.

**Table 4:** Comparison of study subgroups created according to type of hepatectomy (right-lobe LDH vs. left-lobe LDH) type in terms of regeneration-related biochemical parameters.

Variables (Median (95% CI))	Donor Hepatectomy		p *
	Right Lobe	Left Lobe ***	
ACEII Preop	2.2 (1.7–6.4)	1.5 (1.3–2.1)	0.065
ACEII POD1	2.8 (2.2–4.8)	1.6 (1.4–2.0)	0.018
ACEII POD3	2.9 (2.4–6.6)	1.7 (1.4–2.7)	0.010
ACEII POD5	2.8 (2.1–6.1)	1.5 (1.0–3.2)	0.012
ACEII POD7	2.8 (2.1–6.2)	1.1 (0.9–3.1)	0.042
ACEII POD10	3.2 (2.1–5.6)	1.6 (1.2–3.1)	0.058
ACEII POD21	3.7 (1.8–7.2)	1.5 (1.0–4.3)	0.042
p **	0.002	0.994	
AFP Preop	28 (20–61)	13 (12–23)	0.008
AFP POD1	27 (21–60)	15 (14–27)	0.045
AFP POD3	34 (24–64)	20 (18–31)	0.050
AFP POD5	30 (20–69)	15 (12–28)	0.026
AFP POD7	26 (19–87)	13 (11–41)	0.028
AFP POD10	36 (20–80)	17 (13–49)	0.079
AFP POD21	39(19–105)	14 (11–46)	0.046
p **	0.035	0.119	
DCP Preop	9 (7–18)	6 (3–7)	0.010
DCP POD1	11 (8–19)	6(5–6)	0.008
DCP POD3	11 (8–19)	6 (5–10)	0.021
DCP POD5	8 (7–209)	7 (8–14)	0.098
DCP POD7	10 (6–27)	6 (3–11)	0.008
DCP POD10	11 (7–29)	6 (4–14)	0.058
DCP POD21	13 (7–30)	6 (4–18)	0.076
p **	0.129	0.035	
RBP Preop	60 (48–129)	35 (26–49)	0.044
RBP POD1	71 (48–120)	35 (31–61)	0.022
RBP POD3	86 (61–136)	43 (30–61)	0.015
RBP POD5	65 (52–128)	55 (36–93)	0.102
RBP POD7	62 (47–154)	29 (23–80)	0.009
RBP POD10	62 (49–134)	34 (32–89)	0.039
RBP POD21	73 (49–173)	41 (27–119)	0.104
p **	0.335	0.125	
ODC Preop	13 (7–24)	6 (4–12)	0.050
ODC POD1	13 (10–26)	6 (5–17)	0.059
ODC POD3	14 (8–26)	6 (5–18)	0.056
ODC POD5	13 (9–27)	6 (6–12)	0.021
ODC POD7	14 (9–30)	8 (6–15)	0.081
ODC POD10	14 (9–32)	5 (4–13)	0.020
ODC POD21	12 (8–36)	5 (4–12)	0.015
p **	0.001	0.580	

* Mann–Whitney U test; ** Friedman test; *** Left or left-lobe lateral segment.

**Table 5:** Comparison of the study subgroups created according to the postoperative complications status in terms of routine biochemical parameters.

Variables (Median (95% CI))	Postoperative Complications ***		p *
	Yes	No	
AST Preop	22 (16–24)	20 (19–22)	0.748
AST POD1	237 (185–415)	242 (207–267)	0.536
AST POD3	111 (110–162)	97 (90–115)	0.231
AST POD5	75 (45–125)	61 (54–73)	0.215
AST POD7	66 (44–73)	47 (43–55)	0.064
AST POD10	41 (37–47)	37 (35–44)	0.413
AST POD21	37 (26–58)	32 (30–36)	0.243
p **	<0.001	<0.001	
ALT Preop	20 (17–23)	18 (15–20)	0.426
ALT POD1	362 (178–847)	307 (244–358)	0.239
ALT POD3	198 (118–358)	196 (151–244)	0.502
ALT POD5	161 (95–193)	117 (102–137)	0.160
ALT POD7	93 (81–149)	82 (72–94)	0.109
ALT POD10	69 (50–110)	55 (44–70)	0.184
ALT POD21	53 (43–75)	36 (31–41)	0.056
p **	<0.001	<0.001	
GGT Preop	20 (18–29)	17 (15–20)	0.426
GGT POD1	37 (21–46)	21 (17–31)	0.239
GGT POD3	47 (44–107)	36 (29–45)	0.502
GGT POD5	89 (50–110)	65 (58–76)	0.160
GGT POD7	101 (45–104)	72 (64–83)	0.109
GGT POD10	75 (62–104)	66 (59–84)	0.184
GGT POD21	77 (63–142)	61 (58–73)	0.056
p **	<0.001	<0.001	
ALP Preop	62 (58–72)	70 (65–82)	0.457
ALP POD1	65 (60–73)	62 (56–69)	0.302
ALP POD3	75 (66–86)	67 (62–75)	0.457
ALP POD5	86 (70–109)	81 (75–93)	0.542
ALP POD7	86 (68–110)	90 (79–98)	0.975
ALP POD10	93 (74–116)	93 (88–115)	0.719
ALP POD21	108 (81–144)	94 (84–115)	0.571
p **	<0.001	<0.001	
TBil Preop	0.7 (0.3–0.9)	0.5 (0.5–0.6)	0.217
TBil POD1	2.0 (1.8–3.5)	1.6 (1.4–2.1)	0.154
TBil POD3	2.3 (1.3–4.4)	1.8 (1.4–2.5)	0.287
TBil POD5	1.1 (0.7–2.3)	0.9 (0.8–1.2)	0.272
TBil POD7	1.1 (0.6–1.7)	0.6 (0.6–0.8)	0.017
TBil POD10	0.7 (0.5–1.8)	0.5 (0.4–0.7)	0.114
TBil POD21	0.7 (0.5–0.8)	0.5 (0.5–0.6)	0.009
p **	<0.001	<0.001	

* Mann–Whitney U test; ** Friedman test; *** complications requiring relaparotomy.

**Table 6:** Comparison of the study subgroups created according to the postoperative complications status in terms of regeneration-related biochemical parameters.

Variables (Median (95% CI))	Postoperative Complications ***		p *
	Yes	No	
ACEII Preop	1.5 (1.5–12.5)	2.2 (1.8–6.4)	0.585
ACEII POD1	2.2 (1.7–12.5)	2.6 (1.9–4.8)	0.801
ACEII POD3	2.4 (1.5–12.5)	2.5 (2.3–5.4)	0.556
ACEII POD5	2.1 (1.5–12.5)	2.4 (2.1–5.3)	0.556
ACEII POD7	2.2 (1.6–12.5)	2.8 (1.8–4.7)	0.747
ACEII POD10	2.4 (2.1–12.5)	2.7 (1.8–4.3)	0.695
ACEII POD21	1.9 (1.1–12.5)	2.6 (1.6–6.2)	0.713
p **	0.072	0.027	
AFP Preop	20 (17–126)	24 (18–59)	0.801
AFP POD1	22 (14–126)	25 (20–51)	0.780
AFP POD3	32 (13–126)	31 (22–58)	0.547
AFP POD5	22 (16–126)	27 (19–53)	0.856
AFP POD7	18 (118–126)	26 (20–55)	0.476
AFP POD10	16 (13–126)	28 (20–50)	0.510
AFP POD21	14 (12–126)	34 (16–69)	0.444
p **	0.529	0.003	
DCP Preop	5 (3–33)	9 (6–14)	0.275
DCP POD1	6 (4–33)	9 (7–14)	0.356
DCP POD3	6 (5–33)	11 (8–17)	0.146
DCP POD5	6 (5–33)	8 (7–15)	0.300
DCP POD7	6 (4–33)	9 (6–17)	0.213
DCP POD10	7 (3–34)	9 (7–16)	0.467
DCP POD21	5 (4–34)	10 (7–21)	0.298
p **	0.402	0.026	
RBP Preop	57 (48–251)	49 (36–89)	0.510
RBP POD1	48 (38–251)	61 (45–117)	0.812
RBP POD3	64 (43–251)	64 (49–122)	0.989
RBP POD5	51 (42–251)	65 (54–120)	0.664
RBP POD7	53 (36–251)	55 (36–118)	0.944
RBP POD10	57 (49–251)	59 (45–115)	0.758
RBP POD21	65 (50–251)	64 (41–134)	0.526
p **	0.763	0.116	
ODC Preop	6 (2–42)	11 (7–16)	0.245
ODC POD1	6 (5–42)	13 (10–17)	0.281
ODC POD3	7 (5–42)	14 (9–19)	0.233
ODC POD5	6 (6–42)	12 (9–18)	0.555
ODC POD7	6 (5–42)	13 (10–19)	0.493
ODC POD10	6 (4–42)	13 (10–18)	0.449
ODC POD21	6 (5–42)	12 (8–18)	0.445
p **	0.142	0.027	

* Mann–Whitney U test; ** Friedman test; *** complications requiring relaparotomy.

**Table 7:** Comparison of study subgroups created according to sex in terms of routine biochemical parameters.

Variables (Median (95% CI))	Sex		p *
	Female	Male	
AST Preop	18 (17–22)	20 (19–22)	0.123
AST POD1	206 (179–247)	246 (218–313)	0.208
AST POD3	93 (84–111)	111 (95–131)	0.100
AST POD5	65 (53–78)	65 (57–77)	0.803
AST POD7	43 (35–51)	53 (47–58)	0.019
AST POD10	37 (37–45)	42 (37–54)	0.062
AST POD21	32 (24–35)	33 (32–40)	0.035
p **	<0.001	<0.001	
ALT Preop	15 (14–20)	21 (20–30)	0.001
ALT POD1	233 (177–326)	357 (262–408)	0.040
ALT POD3	149 (117–223)	208 (172–258)	0.064
ALT POD5	108 (80–137)	126 (111–156)	0.137
ALT POD7	71 (54–85)	91 (85–111)	0.012
ALT POD10	44 (40–56)	70 (61–88)	0.005
ALT POD21	27 (23–32)	48 (38–57)	<0.001
p **	<0.001	<0.001	
GGT Preop	15 (12–18)	20 (18–27)	0.001
GGT POD1	17 (16–34)	27 (21–36)	0.086
GGT POD3	36 (24–47)	41 (32–54)	0.319
GGT POD5	65 (54–76)	69 (60–84)	0.240
GGT POD7	67 (57–77)	82 (72–98)	0.078
GGT POD10	66 (59–92)	69 (62–94)	0.262
GGT POD21	61 (49–67)	71 (60–89)	0.057
p **	<0.001	<0.001	
ALP Preop	64 (59–72)	72 (65–83)	0.148
ALP POD1	57 (52–64)	65 (62–72)	0.098
ALP POD3	73 (62–80)	67 (62–73)	0.740
ALP POD5	88 (77–99)	77 (70–92)	0.459
ALP POD7	94 (86–100)	84 (75–99)	0.668
ALP POD10	102 (88–127)	92 (82–101)	0.394
ALP POD21	94 (78–123)	94 (87–123)	0.263
p **	<0.001	<0.001	
TBil Preop	0.5 (0.5–0.7)	0.5(0.5–0.6)	0.093
TBil POD1	1.7 (1.4–1.9)	1.85(1.4–2.4)	0.515
TBil POD3	1.8 (1.4–2.3)	2(1.4–2.7)	0.709
TBil POD5	0.9 (0.8–1.3)	0.9(0.7–1.3)	0.787
TBil POD7	0.7 (0.6–0.8)	0.6(0.6–0.9)	0.568
TBil POD10	0.5 (0.4–0.7)	0.5(0.5–0.8)	0.682
TBil POD21	0.6 (0.5–0.6)	0.5(0.5–0.6)	0.154
p **	<0.001	<0.001	

* Mann–Whitney U test; ** Friedman test.

**Table 8:** Comparison of study subgroups created according to sex in terms of regeneration-related biochemical parameters.

Variables (Median (95% CI))	Sex		p *
	Female	Male	
ACEII Preop	1.7 (1.4–6.4)	2.2 (2.0–5.1)	0.418
ACEII POD1	2.0 (1.7–4.5)	2.6 (2.2–10.1)	0.552
ACEII POD3	2.4 (2.1–6.7)	2.8 (2.4–4.9)	0.482
ACEII POD5	2.2 (1.8–6.1)	2.8 (2.0–5.3)	0.520
ACEII POD7	2.2 (1.6–6.8)	2.8 (2.2–4.8)	0.329
ACEII POD10	2.4 (1.5–5.6)	2.8 (2.1–4.3)	0.675
ACEII POD21	1.9 (1.4–6.4)	3.7 (1.7–7.9)	0.493
*p* **	0.095	0.027	
AFP Preop	21 (15–61)	24 (18–48)	0.514
AFP POD1	27 (18–51)	21 (20–55)	0.938
AFP POD3	24 (18–69)	32 (25–49)	0.325
AFP POD5	22 (17–61)	27 (20–49)	0.489
AFP POD7	21 (13–66)	22 (19–57)	0.402
AFP POD10	21 (16–80)	28 (20–56)	0.385
AFP POD21	20 (14–93)	34 (19–105)	0.382
*p* **	0.085	0.025	
DCP Preop	6 (4–14)	9 (7–15)	0.182
DCP POD1	6 (6–17)	10 (8–16)	0.216
DCP POD3	9 (6–17)	11 (9–19)	0.355
DCP POD5	7 (6–19)	8 (7–15)	0.793
DCP POD7	6 (5–19)	9 (6–18)	0.202
DCP POD10	8 (6–29)	9 (7–15)	0.612
DCP POD21	7 (5–30)	13 (7–28)	0.396
*p* **	0.004	0.260	
RBP Preop	48 (31–129)	56 (42–82)	0.441
RBP POD1	61 (42–102)	53 (46–120)	0.489
RBP POD3	61 (44–134)	78 (57–115)	0.605
RBP POD5	55 (44–128)	65 (61–120)	0.344
RBP POD7	47 (36–126)	70 (46–139)	0.441
RBP POD10	49 (39–134)	62 (49–89)	0.458
RBP POD21	52 (41–159)	73 (50–134)	0.930
*p* **	0.127	0.634	
ODC Preop	10 (5–16)	11 (6–24)	0.459
ODC POD1	12 (5–20)	12 (9–17)	0.315
ODC POD3	12 (6–19)	13 (8–19)	0.482
ODC POD5	12 (6–23)	12 (9–18)	0.488
ODC POD7	13 (6–24)	11 (9–19)	0.559
ODC POD10	10 (5–32)	13 (9–17)	0.532
ODC POD21	11 (6–36)	12 (8–37)	0.641
*p* **	0.015	0.133	

* Mann–Whitney U test; ** Friedman test.

**Table 9:** Comparison of the study subgroups created according to the percentage of remnant liver volume in terms of regeneration-related biochemical parameters.

Variables (Median (95% CI))	Remnant Liver Volume (%)			p *
	≤30	31–35	≥36	
ACEII Preop	2.2 (1.4–6.6)	4.3 (1.7–7.2)	1.5 (1.3–2.1)	0.062
ACEII POD1	2.6 (2.3–4.5)	3.7 (2.0–8.9)	1.6 (1.4–2.0)	0.029
ACEII POD3	2.7 (2.4–5.1)	3.9 (2.3–9.4)	1.7 (1.4–2.7)	0.014
ACEII POD5	2.4 (2.1–5.1)	4.2 (2.1–9.1)	1.5 (1.0–3.2)	0.014
ACEII POD7	2.7 (1.6–4.5)	4.0 (1.9–8.6)	1.1 (0.9–3.1)	0.031
ACEII POD10	2.7 (1.8–5.6)	3.8 (2.4–9.9)	1.6 (1.2–3.1)	0.042
ACEII POD21	2.6 (1.5–7.9)	5.6 (1.6–12.5)	1.5 (1.0–4.3)	0.040
p **	0.470	0.009	0.994	
AFP Preop	26 (14–59)	34 (21–103)	13 (12–23)	0.005
AFP POD1	26 (19–51)	31 (21–93)	15 (14–27)	0.039
AFP POD3	31 (18–64)	37 (24–98)	20 (18–31)	0.039
AFP POD5	29 (18–61)	33 (21–77)	15 (12–28)	0.034
AFP POD7	24 (19–55)	36 (18–111)	13 (11–41)	0.041
AFP POD10	31 (18–50)	36 (20–91)	17 (13–49)	0.064
AFP POD21	39 (19–126)	46 (14–126)	14 (11–46)	0.062
p **	0.108	0.132	0.119	
DCP Preop	8 (4–14)	12 (8–30)	6 (3–7)	0.009
DCP POD1	10 (7–17)	13 (8–29)	6 (5–6)	0.010
DCP POD3	11 (8–17)	14 (7–27)	6 (5–10)	0.029
DCP POD5	8 (6–15)	14 (7–26)	7 (7–14)	0.070
DCP POD7	9 (6–16)	12 (6–30)	6 (3–11)	0.008
DCP POD10	9 (7–17)	12 (7–30)	6 (4–14)	0.048
DCP POD21	10 (6–34)	15 (7–33)	6 (4–18)	0.072
p **	0.106	0.586	0.035	
RBP Preop	50 (22–89)	76 (54–190)	35 (26–49)	0.013
RBP POD1	68 (42–100)	93(46–175)	35 (31–61)	0.032
RBP POD3	82 (44–122)	113 (62–174)	43 (30–61)	0.015
RBP POD5	65 (46–107)	80 (51–197)	55 (36–93)	0.088
RBP POD7	58 (36–99)	88 (53–196)	29 (23–80)	0.011
RBP POD10	56 (45–134)	65 (52–224)	34 (32–89)	0.064
RBP POD21	69 (36–136)	79 (49–251)	41 (27–119)	0.109
p **	0.059	0.658	0.125	
ODC Preop	10 (5–16)	14 (10–33)	6 (4–12)	0.042
ODC POD1	11 (5–20)	14 (10–37)	6 (5–17)	0.050
ODC POD3	11 (8–19)	15 (8–42)	6 (5–18)	0.041
ODC POD5	13 (8–23)	15 (10–41)	6 (6–12)	0.027
ODC POD7	12 (7–19)	15 (9–35)	8 (6–15)	0.076
ODC POD10	15 (6–23)	14 (9–38)	5 (4–13)	0.020
ODC POD21	15 (7–42)	12 (7–41)	5 (4–12)	0.019
p **	0.011	0.042	0.580	

* Kruskal–Wallis test; ** Friedman test.

## Data Availability

The datasets analyzed during the current study are available from the corresponding author upon reasonable request.

## References

[1] Kiseleva Y.V., Antonyan S.Z., Zharikova T.S., Tupikin K.A., Kalinin D.V., Zharikov Y.O. (2021). Molecular pathways of liver regeneration: A comprehensive review. World. J. Hepatol..

[2] Michalopoulos G.K., Bhushan B. (2021). Liver regeneration: Biological and pathological mechanisms and implications. Nat. Rev. Gastroenterol. Hepatol..

[3] Gilgenkrantz H., Collin de l’Hortet A. (2018). Understanding Liver Regeneration: From Mechanisms to Regenerative Medicine. Am. J. Pathol..

[4] Lopez-Luque J., Fabregat I. (2018). Revisiting the liver: From development to regeneration—what we ought to know!. Int. J. Dev. Biol..

[5] Wirth K.M., Kizy S., Steer C.J. (2018). Liver Regeneration in the Acute Liver Failure Patient. Clin. Liver Dis..

[6] Guan G.W., Gao L., Wang J.W., Wen X.J., Mao T.H., Peng S.W., Zhang T., Chen X.M., Lu F.M. (2020). Exploring the mechanism of liver enzyme abnormalities in patients with novel coronavirus-infected pneumonia. Zhonghua Gan Zang Bing Za Zhi.

[7] Guan W.J., Ni Z.Y., Hu Y., Liang W.H., Ou C.Q., He J.X., Liu L., Shan H., Lei C.L., Hui D.S.C. (2020). Clinical Characteristics of Coronavirus Disease 2019 in China. N. Engl. J. Med..

[8] Li J., Fan J.G. (2020). Characteristics and Mechanism of Liver Injury in 2019 Coronavirus Disease. J. Clin. Transl. Hepatol..

[9] Sahin T.T., Akbulut S., Yilmaz S. (2020). COVID-19 pandemic: Its impact on liver disease and liver transplantation. World J. Gastroenterol..

[10] Francisco V., Sanz M.J., Real J.T., Marques P., Capuozzo M., Ait Eldjoudi D., Gualillo O. (2022). Adipokines in Non-Alcoholic Fatty Liver Disease: Are We on the Road toward New Biomarkers and Therapeutic Targets?. Biology.

[11] Sanchez-Sevilla L., Mendieta-Condado E., Hernandez-Munoz R. (2016). Putrescine treatment reverses alpha-tocopherol-induced desynchronization of polyamine and retinoid metabolism during rat liver regeneration. J. Transl. Med..

[12] Kuhlmann W.D., Peschke P. (2006). Hepatic progenitor cells, stem cells, and AFP expression in models of liver injury. Int. J. Exp. Pathol..

[13] Feng H., Li B., Li Z., Wei Q., Ren L. (2021). PIVKA-II serves as a potential biomarker that complements AFP for the diagnosis of hepatocellular carcinoma. BMC Cancer.

[14] Miyaoka Y., Miyajima A. (2013). To divide or not to divide: Revisiting liver regeneration. Cell Div..

[15] Yilmaz S., Akbulut S., Usta S., Ozsay O., Sahin T.T., Sarici K.B., Karabulut E., Baskiran A., Gonultas F., Ozdemir F. (2021). Diagnostic and therapeutic management algorithm for biliary complications in living liver donors. Transpl. Int..

[16] Eshkenazy R., Dreznik Y., Lahat E., Zakai B.B., Zendel A., Ariche A. (2014). Small for size liver remnant following resection: Prevention and management. Hepatobiliary Surg. Nutr..

[17] Guglielmi A., Ruzzenente A., Conci S., Valdegamberi A., Iacono C. (2012). How much remnant is enough in liver resection?. Dig. Surg..

[18] Horn K.D., Wax P., Schneider S.M., Martin T.G., Nine J.S., Moraca M.A., Virji M.A., Aronica P.A., Rao K.N. (1999). Biomarkers of liver regeneration allow early prediction of hepatic recovery after acute necrosis. Am. J. Clin. Pathol..

[19] O’Grady J.G., Alexander G.J., Hayllar K.M., Williams R. (1989). Early indicators of prognosis in fulminant hepatic failure. Gastroenterology.

[20] Mohapatra N., Sinha P.K., Sasturkar S.V., Patidar Y., Pamecha V. (2020). Preoperative Alanine Aminotransferase and Remnant Liver Volume Predict Liver Regeneration After Live Donor Hepatectomy. J. Gastrointest. Surg..

[21] Sahin T.T., Sarici K.B., Kilci B., Koc C., Otan E., Karakas S., Kutluturk K., Aydin C., Kayaalp C., Yilmaz S. (2019). Factors Influencing Mortality Following Liver Transplant in Acute Liver Failure: A Single Center Experience. Exp. Clin. Transplant.

[22] Campani C., Bamba-Funck J., Campion B., Sidali S., Blaise L., Ganne-Carrie N., Demory A., Sutter O., Larrey E., Evain M. (2022). Baseline ALBI score and early variation of serum AFP predicts outcomes in patients with HCC treated by atezolizumab-bevacizumab. Liver Int..

[23] Farmer D.G., Anselmo D.M., Ghobrial R.M., Yersiz H., McDiarmid S.V., Cao C., Weaver M., Figueroa J., Khan K., Vargas J. (2003). Liver transplantation for fulminant hepatic failure: Experience with more than 200 patients over a 17-year period. Ann. Surg..

[24] Rao K.N., Virji M.A., Moraca M.A., Diven W.F., Martin T.G., Schneider S.M. (1993). Role of serum markers for liver function and liver regeneration in the management of chloroform poisoning. J. Anal. Toxicol..

[25] Zuckerman J., Gorgen A., Acuna S.A., Abreu P., Goldaracena N., Galvin Z., Cattral M.S., Ghanekar A., McGilvray I.D., Lilly L.B. (2021). Outcomes of Highly Selected Live Donors With a Future Liver Remnant Less Than or Equal to 30%: A Matched Cohort Study. Transplantation.

[26] Pamecha V., Patil N.S., Parthasarathy K., Sinha P.K., Mohapatra N., Rastogi A., Rudrakumar K., Mukund A., Chaudhary A., Kanal U. (2022). Expanding donor pool for live donor liver transplantation: Utilization of donors with non-alcoholic steatohepatitis after optimization. Langenbecks Arch. Surg..

[27] Fernandez-Atucha A., Izagirre A., Fraile-Bermudez A.B., Kortajarena M., Larrinaga G., Martinez-Lage P., Echevarria E., Gil J. (2017). Sex differences in the aging pattern of renin-angiotensin system serum peptidases. Biol. Sex Differ..

[28] Salah H.M., Mehta J.L. (2021). Hypothesis: Sex-Related Differences in ACE2 Activity May Contribute to Higher Mortality in Men Versus Women With COVID-19. J Cardiovasc. Pharmacol. Ther..

[29] Bizzaro D., Crescenzi M., Di Liddo R., Arcidiacono D., Cappon A., Bertalot T., Amodio V., Tasso A., Stefani A., Bertazzo V. (2018). Sex-dependent differences in inflammatory responses during liver regeneration in a murine model of acute liver injury. Clin. Sci..

[30] Marcos R., Correia-Gomes C., Miranda H., Carneiro F. (2015). Liver gender dimorphism--insights from quantitative morphology. Histol. Histopathol..

[31] Sutti S., Tacke F. (2018). Liver inflammation and regeneration in drug-induced liver injury: Sex matters!. Clin. Sci..

